# Sequencing and bioinformatics analysis of exosome-derived miRNAs in mouse models of pancreatic injury induced by OSA

**DOI:** 10.3389/fphys.2025.1712442

**Published:** 2025-11-11

**Authors:** Jiuhuang Lan, Jiayi Lin, Yaopeng Guo, Meilin Ji, Yizhao Lin, Qingshi Chen

**Affiliations:** 1 The Second Clinical Medical College, Fujian Medical University, Quanzhou, China; 2 The Second Affiliated Hospital of Fujian Medical University, Quanzhou, China; 3 Department of Endocrinology and Metabolism, The Second Affiliated Hospital of Fujian Medical University, Quanzhou, China; 4 Department of Cardiology, Gutian County Hospital, Gutian, China

**Keywords:** exosome-derived miRNAs, obstructive sleep apnea, pancreatic injury, chronic intermittent hypoxia, bioinformatics analysis

## Abstract

**Introduction:**

The role of exosomal miRNAs involved in the pathogenesis and development of pancreatic injury caused by obstructive sleep apnea (OSA) remains unclear. Here, miRNA sequencing (miRNA-seq) was used to investigate the expression profile of exosomal miRNAs in pancreatic tissue in a mouse model of chronic intermittent hypoxia (CIH) -induced pancreatic injury.

**Methods:**

Firstly, exosomes were isolated from pancreatic tissues of mice subjected to chronic intermittent hypoxia (CIH) or control conditions. Then, miRNA sequencing was performed, and differential expression analysis identified DE miRNAs. The potential functions of target genes were predicted by GO and KEGG analyses. Finally, a ceRNA interaction network was constructed to illustrate the relationships between miRNAs and targeted mRNAs.

**Results:**

A total of 55 newly predicted miRNAs and 277 existing miRNAs were identified in pancreatic tissue exosomes from the mouse model of chronic intermittent hypoxic-induced pancreatic injury. 3 upregulated and 33 downregulated miRNAs with differential expression were identified. 8 exosome-derived miRNAs were selected to construct CNC networks to predict target genes. Some targets and pathways were elucidated by GO analysis and KEGG analysis.

**Conclusion:**

Our work first comprehensively studied the expression profile of exosome-derived miRNA in pancreatic tissue of mouse models of OSA-induced pancreatic injury, bringing fresh perspectives on managing diabetes mellitus brought on by OSA.

## Introduction

As a prevalent chronic sleep disorder, obstructive sleep apnea (OSA) is characterized by chronic intermittent hypoxia (CIH). Multiple health impairments, including lung damage (Tuleta et al., 2016), renal injury (Zhao et al., 2021), metabolic disorders (Almendros et al., 2022), and cardiovascular disease (Chen et al., 2021), can arise from long-term untreated OSA. These conditions decrease quality of life and place a financial and psychological strain on patients. Moreover, the impairment of glucose metabolism and OSA are tightly associated. OSA is advised as a stand-alone risk factor for type 2 diabetes mellitus (Abud et al., 2019; Gabryelska et al., 2021). According to earlier findings, rats exposed to CIH may develop pancreatic damage and insulin secretion disorders as a result of OSA (Wang et al., 2017). In the meantime, CIH encourages high levels of reactive oxygen species generation, resulting in glucose intolerance and insulin resistance. Nevertheless, there are still relatively few research examining how OSA affects insulin, pancreatic cells, and diabetes, and the underlying molecular process is still unknown. Zeng, S., et al. have shown that OSA contributes to impaired glucose metabolism primarily through chronic intermittent hypoxia (CIH) and sleep fragmentation, which activate sympathetic nervous system activity and promote systemic inflammation and oxidative stress (Zeng et al., 2024). These processes can lead to insulin resistance and β-cell dysfunction, thereby increasing the risk of type 2 diabetes mellitus.

Exosomes are small extracellular vesicles that have a diameter of 30–150 nm with a double-layer lipid membrane structure. Mammals secrete them, and they can be found in blood, cell fluid, breast milk, urine, and other bodily fluids (Yang et al., 2020). Although they were treated as cellular waste products when they were first discovered, studies are now gradually showing that they are critical mediators of intercellular communication in various pathological and physiological processes (He et al., 2021; Whitford and Guterstam, 2019). Additionally, proteins, lipids, microRNAs (miRNAs), and other RNAs are carried by exosomes. It has been proposed that miRNAs, which are engaged in a variety of physiological and pathological processes, are responsible for a number of exosome biological effects (Mori et al., 2019; Hu et al., 2020; Isaac et al., 2021). Exosomal miRNAs are emerging as key regulators in the development of diabetes mellitus, primarily in terms of insulin resistance and pancreatic β-cell damage (Hu et al., 2021). Recent studies have shown that exosomes from pancreatic β-cells containing the miR-29 family (miR-29s) play a crucial role in modulating liver insulin sensitivity and glucose regulation. In particular, exosomes derived from the islets that carry miR-29s interact with insulin signaling pathways in the liver, thereby diminishing hepatic insulin sensitivity. (Li et al., 2021). Other studies have suggested that in diabetes mellitus, β-cell dysfunction or damage may be related to the enrichment of exosome-specific miRNA (Seyhan et al., 2016; Liu et al., 2020; Khan et al., 2022). Nonetheless, limited research has explored the changes in exosomal miRNAs within a pancreas affected by OSA.

In our work, exosomal miRNAs were extracted from pancreatic tissue in the mouse model and control group. Then the two groups of exosomal miRNAs were sequenced. After the miRNAs were quantitatively analyzed, the differentially expressed (DE) miRNAs were screened, the miRNA target genes (TGs) were predicted, and the DE miRNA TGs were functionally annotated and enriched. In addition, we predicted novel miRNAs and obtained their partial sequences. Collectively, our research is the first to uncover the expression patterns and possible functions of modified exosomal miRNAs in the pancreases of mice subjected to CIH. These findings could provide new targets for the clinical treatment of diabetes mellitus caused by OSA in humans.

## Methods

### Preparation of animal models

In this work, BALB/c mice from Weitong Lihua in Beijing, China were employed. The license for the use of experimental animals is SCXK (MIN) 2022–0001, while the production license is SCXK (JING) 2021–0006. The research protocol was approved by the Research Ethics Committee of Fujian Medical University’s Second Affiliated Hospital. All mice were fed with unlimited access to water and food.

We used our previously published approach to establish the CIH model (Chen et al., 2019). Simply put, we took 18 mice mentioned above and randomly divided them into 2 groups. There were 9 mice in the CIH group and 9 mice in the control group. During the modeling period of 8 weeks, a small number of mice in the CIH group succumbed to the severe hypoxic stress. These deceased mice were promptly excluded, and the cohort was replenished to maintain an adequate sample size for the protocol. From the final surviving cohorts in each group, 3 mice were randomly selected for the subsequent experiments. All the mice that were allocated to the CIH group were kept in specially designed, commercially available chambers (ProOx-100-CIH-M, Shanghai TOW Intelligent Technology Co., Ltd.). The system pumps pure nitrogen into the chamber and reduces O_2_ to 6% in 60 s. Then, the control system permits rapid oxygen replenishment, which causes O_2_ to reoxidize quickly to 21% in 60 s. For a total of 8 weeks, this 2-min CIH cycle is repeated thirty times every hour for eight hours each day. In the control group, the tube of the experimental device was directly connected to the indoor air during the entire experiment. Following 8 weeks of exposure to CIH, all mice were put to sleep with isoflurane anesthesia and then euthanized by cervical dislocation performed by a qualified practitioner. After that, the tissues of the pancreas were harvested and sectioned, with all assessments performed in a blinded manner by two experienced pathologists.

### Exosome isolation and RNA extraction

Following a 5-min 500×g centrifugation of the liquid sample, the cells were extracted from the sample. Then, the supernatant was centrifuged for 10 min at 2000×g after being transferred to another polycarbonate tube. Next, the shed microvesicles were removed by centrifuging at 10,000×g for 30 min. Using a 0.22 μm membrane filter, the centrifuged supernatant from centrifugation was filtered and centrifuged at 100,000×g for 2 h. Exosome particles were washed once in 1×PBS, then re-suspended in 1×PBS, and kept for subsequent use at −80 °C. The isolated vesicles were identified as exosomes based on their cup-shaped morphology observed under transmission electron microscopy and their size distribution as determined by nanoparticle tracking analysis. And exosomes were isolated from pancreatic tissue homogenates using differential ultracentrifugation, a widely established method for enriching extracellular vesicles from biological samples (Bashir et al., 2025).

TRI Reagent (Sigma: T9424) was used to extract RNA from the samples. Through the use of denatured agar-gel electrophoresis and ultraviolet-ultraviolet spectrophotometry (NanoDrop® ND-1000), the purity, concentration, and integrity of RNA samples for transcriptomic sequencing were certified.

### Electron microscopy

The exosomes’ morphology was examined using Tecnai G2 Spirit 120 KV transmission electron microscopy. In brief, PBS was used to dilute the suspended exosomes before each sample was placed on a copper grid coated with carbon. After letting the exosomes precipitate for 10 minutes, airflow mesh paper was used to adsorber the residual solution. After immersing the mesh in 3% glutaraldehyde for 5 minutes at room temperature, it was washed ten times with deionized water. After that, the copper grid was exposed to a 4% uranyl acetate solution for 10 min at room temperature and a 1% methylcellulose solution for 5 min at the same temperature. In 30 min, the samples were dried and inspected under a transmission electron microscope.

### miRNA sequencing and data analysis

Using miRBase, reads from the reference genome were compared to mature sequences of known miRNAs with a maximum of one mismatch allowed. The range of matches was 2 nt upstream and 5 nt downstream. The readings that were found were regarded as known miRNAs. Based on the biological characteristics of miRNA, the potential precursor sequences were found by the Illumina Genome Analyzer IIx program by comparing readings with genome location data. RNA-fold (version 2.1.7) and the read distribution on the precursor sequence were used to evaluate energy information for the precursor structure using a Bayesian model (Najeh et al., 2021). This model took into account features related to miRNA synthesis, mature sequence characteristics, star sequences, and loops, and consequently predicted novel miRNA. Each sample’s miRNA expression levels were counted, and the transcript per million (TPM) approach was then used to standardize the result (Zhao et al., 2020).

DESeq2 is suitable for biological repetition studies, whereas differential expression analysis between sample groups may be utilized to acquire DE miRNA sets between two biological scenarios (McDermaid et al., 2019). DE miRNAs between two samples can be identified in research without biological replication by using EdgeR (Liu et al., 2021). In our current study, DESeq2 was used to compare the relative expression levels of the two (groups) samples, and the DE miRNAs were categorized as either upregulated or downregulated. The fold change (FC) and P value in expression were used to determine the screening threshold for the identification of DE miRNAs, which was as follows: |log2 (FC)|≥2, P-value <0.05. To lower the false discovery rate (FDR) and correct the P values used in the identification of DE miRNAs, the Benjamini Hochberg technique was applied.

### GO annotation and KEGG pathway enrichment analysis

For the target genes identified by exosome-derived miRNAs, we carried out KEGG pathway enrichment analysis and GO analysis to get more insight into the biological functions of the genes. The categories of biological process (BP), molecular function (MF), and cellular component (CC) were present. The important routes all have a p-value of less than 0.05.

### Construction of a ceRNA interaction network

To construct the miRNA-mRNA network, 8 significantly differentially expressed miRNAs were chosen using Arraystar’s in-house miRNA target prediction software, which was developed using TargetScan and miRanda. Next, we constructed a competitive endogenous RNA (ceRNA) network using Cytoscape 2.8.3 with eight selected miRNAs and targeted genes. Pearson’s correlation coefficient was set at (r) > 0.97.

### qPCR

The quantitative PCR (qPCR) procedure was performed following the MIQE guidelines, using well-validated protocols for miRNA quantification (Bustin, 2024). The qPCR primers were synthesized by Shanghai Aksomics Biotechnology Co., Ltd., and their sequences are displayed in Table1. QuantStudio5 Real-time PCR system was used to prepare the qPCR reaction system. The raw materials included forward and reverse primers and cDNA templates, and the above materials were added into the PCR reaction tube and thoroughly mixed. 5µL of 2X PCR master mix (Arraystar: AS-MR-006–5), 0.5 µL of forward primer (10 µM), 0.5 µL of reverse primer (10 µM), and 8 µL of nuclease-free water made up the reaction system. The steps are as follows: the PCR reaction was performed at 95 °C for 10 min. Bow through 40 PCR cycles, with each cycle consisting of 10 s of denaturation at 95 °C and 60 s of annealing at 60 °C. Lastly, 1 cycle of melting curve analysis cycle was run for 10 s at 95 °C, 60 s for 60 °C, and 15 s for 95 °C. The miRNA enrichment was detected by qPCR using 2^-△△^^Ct^ technique. Quantitative PCR was performed for each cDNA sample in technical triplicate.

**TABLE 1:** Primers used for qPCR.

Genes	Sequence	Tm (°C)	Lengths (bp)
U6	F: 5′GCTTCGGCAGCACATATACTAAAAT3′ R: 5′CGCTTCACGAATTTGCGTGTCAT3′	60	89
mmu-miR-181d-5p	GSP: 5′ GGGGCATTCATTGTTGTCG3′ R: 5′ GTGCGTGTCGTGGAGTCG3′	60	63
mmu-miR-217-5p	GSP: 5′GGGGATACTGCATCAGGAACT3′ R: 5′ GTGCGTGTCGTGGAGTCG3′	60	86
mmu-miR-96-5p	GGTTTGGCACTAGCACAT R: 5′CAGTGCGTGTCGTGGAGT3′	60	66
mmu-miR-5119	GSP: 5′GGGGGTCATCTCATCCTGG3′ R: 5′GTGCGTGTCGTGGAGTCG3′	60	63
mmu-let-7b-3p	GSP:5′GGGGGCTATACAACCTACTGC3′ R:5′GTGCGTGTCGTGGAGTCG3′	60	65
mmu-miR-382-5p	GSP:5′GGGGAAGTTGTTCGTGGTG3′ R: 5′GTGCGTGTCGTGGAGTCG3′	60	63
mmu-miR-484	GSP:5′GAATCAGGCTCAGTCCCC3′ R: 5′CAGTGCGTGTCGTGGAGT3′	60	65
mmu-miR-744-5p	GSP: 5′ATGCGGGGCTAGGGCT3′ R:5′CAGTGCGTGTCGTGGAGT3′	60	61

GSP, Specific primer corresponding to miRNA; R, Primers that match RT primers.

### Methods of statistical analysis

Using IBM SPSS 22.0, the chi-square test was employed to compare the fraction of exosomes with no annotation. The Bonferroni’s test was conducted to compare exosome concentration, relative protein levels, and relative mRNA expression between the two groups. The derived P-values were corrected using the Benjamini–Hochberg method for decreasing the FDR. DESeq2 was used to classify exosome-derived miRNAs with |log2(FC)|≥2; P-value <0.05 as DE miRNAs.

## Results

### Observation of exosomes from pancreatic cells

**FIGURE 1:**
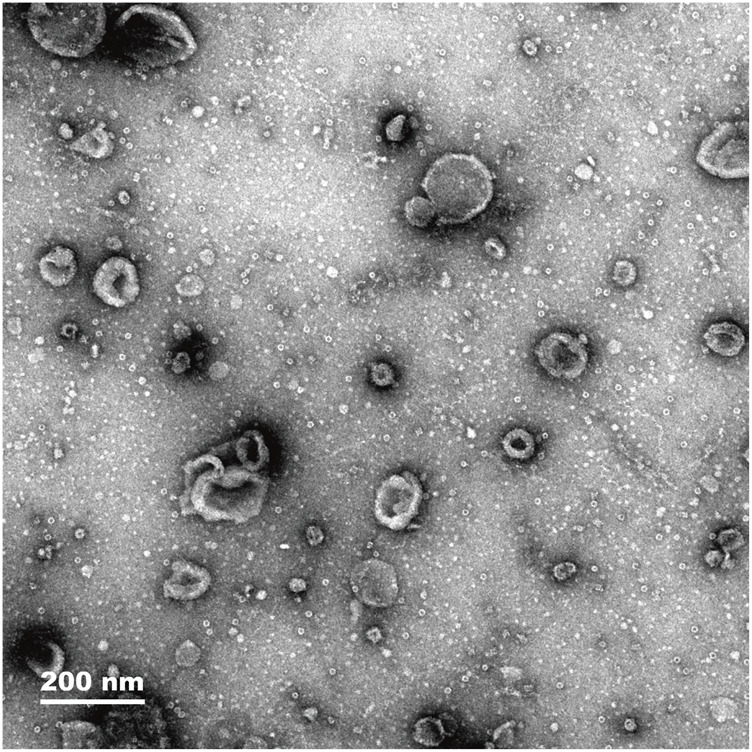
Electron microscopy images showing exosome density of pancreas tissue (magnification, 200 nm).

The histological characteristics of exosomes from mouse pancreatic cells were observed. Exosomes isolated from mouse pancreatic tissue were found to have a cup-shaped form using electron microscopy (Figure 1). According to the NTA results, the median diameter of exosomes isolated from pancreatic tissue was 100 nm. In addition, the characterization of specific exosomal protein markers (e.g., CD63, CD81, TSG101) and a direct comparative analysis of exosomes from both groups were not performed in this preliminary study, which are limitations that future work will address.

### sRNA classification and DE miRNAs

**FIGURE 2:**
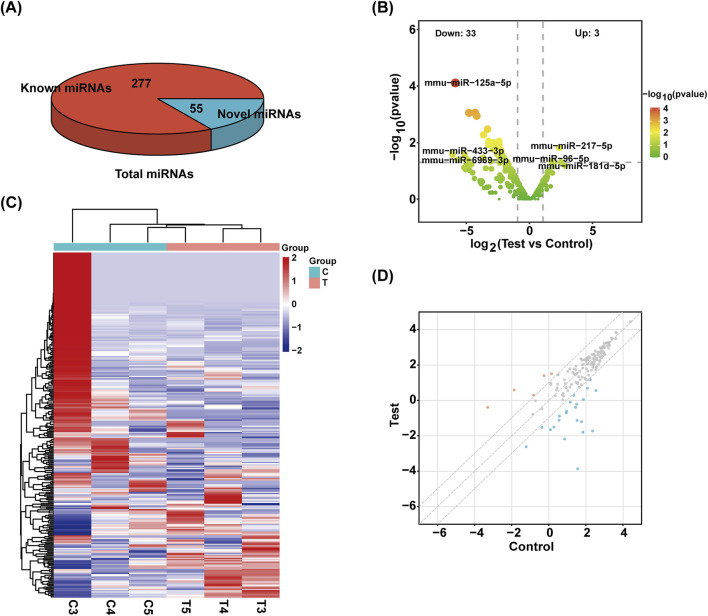
Identification of sRNA. **(A)** The total number of miRNAs identified and the number of known and unknown miRNAs. **(B)** Volcanic map of miRNAs. The samples were classified according to the similarity of different expression levels or quantities and the close relationship between them (|FC| ≥ 2, P-value <0.05). **(C)** The heat maps of different miRNAs are grouped hierarchically. Red means up, blue means down. **(D)** Scatter plot of miRNAs. The values on the X and Y-axes represent the fold change of normalized signal values for each group (|FC| ≥ 2).

Exosomes have been found to include a variety of sRNA types. Using a range of databases, clean reads were filtered using the Bowtie tool, which eliminates non-coding RNAs (ncRNAs) such rRNA, tRNA, snRNA, and snoRNA and repeats to produce unannotated reads that contain miRNAs. The percentage of unlabeled reads containing miRNAs in total sRNAs isolated from mouse pancreatic tissue exosomes was 16.57% (Figure 2A).

Overall, 332 miRNAs were found by sRNA sequencing of mouse pancreatic exosomes, comprising 277 known miRNAs and 55 newly predicted miRNAs. Table 2 demonstrated the top 10 newly predicted miRNAs with high miRDeep2 Score expression. Using |log2 (FC)|≥2; P < 0.05 as references, 3 upregulated miRNAs and 33 downregulated miRNAs were found among the known miRNAs. mmu-miR-181d-5p, mmu-miR-217-5p and mmu-miR-96-5p were the upregulated miRNAs. mmu-miR-433-3p, mmu-miR-6969-3p, mmu-miR-125a-5p, mmu-miR-5119, mmu-miR-5126, mmu-miR-410-3p, mmu-miR-744-5p, mmu-miR-125b-5p, mmu-let-7d-3p and mmu-miR-128-3p were the top 10 DE miRNAs in downregulated miRNAs. mmu-miR-96-5p has been proven to regulate the INS/AKT axis *PIK3R1*, *PRKCE*, *AKT1*, *AKT2*, and *AKT3* genes to promote the occurrence and development of diabetic retinopathy (DR) (Zolfaghari et al., 2023).

**TABLE 2:** The top 10 newly predicted miRNAs with high miRDeep2 Score expression.

Identity	miRDeep2 score	Total RC	Mature sequence	Star sequence
chr13_15097	17.6	32	ggggguguagcucagugguagagc	gaggccccggguucaauccccggc
chr13_15099	13.9	32	ggggguguagcucagugguagagc	gaggccccggguucaauccccggc
chr9_10595	3.2	15	ucugugggaaggaacuacaagacag	acuugugguuuuacuugacuca
chr7_8611	3.2	47	ccccggagaaggcgccug	ggcgcggccggacggga
chr3_3384	3.1	18	cgcggguggggccgggggu	cagccgccgcugggcgca
chr5_6806	2.6	1578	guccugggguggacuga	ggggccccagugggcag
chr2_1668	2.5	26	aucucgcuggggccucc	gggcccaaguguugagaa
chr5_6507	2.3	1193	aucucuuggcucuggccu	gaaauacagggugaaagugauag
chr6_7970	2.2	389	cccagggcuggacugag	aagccuagaguccuggguu
chr6_7371	2.2	128	cacugguaagggcccuggg	uagggcagaggccaaugag

A volcano plot was used to display the statistical significance of the difference between the two groups’ miRNA expression levels (Figure 2B). Using a hierarchical clustering analysis, the DE miRNAs were sorted into groups based on similar or identical expression patterns (Figure 2C). Additionally, a scatter plot was created to visually represent the overall distribution of expression levels and differential multiples of DE miRNAs in both groups (Figure 2D).

### Forecasting and labeling of miRNA target genes

**FIGURE 3:**
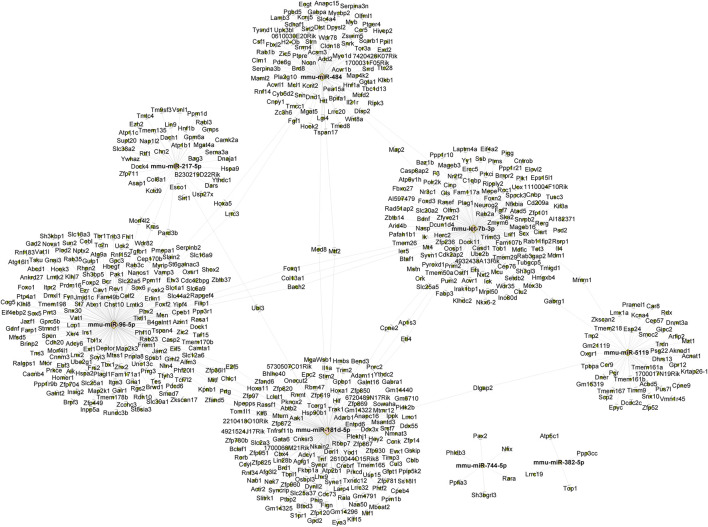
The construction of a miRNA-mRNA network for eight candidate miRNAs. The construction of a miRNA-mRNA network for eight candidate miRNAs. Pink circles represent miRNA and green circles represent targeted genes.

We employed miRanda and Targetscan on 8 exosome-derived miRNAs (mmu-miR-181d-5p, mmu-miR-217-5p, mmu-miR-96-5p, mmu-miR-5119, mmu-let-7b-3p, mmu-miR-382-5p, mmu-miR-484 and mmu-miR-744-5p) based on the gene sequencing data of currently known and recently discovered miRNAs and related species. Annotation data were available for 3,817 TGs. Then, Cytoscape 2.8.3 was used to draw a map of the network. Figure 3 displays the anticipated TGs of each miRNA.

### The biological function prediction of DE miRNA targeted-genes

For every sequenced exosome-derived miRNA, we predicted its target gene and subsequently carried out GO analysis. Through GO analysis, enriched biological processes, molecular roles, and cellular components for the DE miRNAs were identified.

**FIGURE 4:**
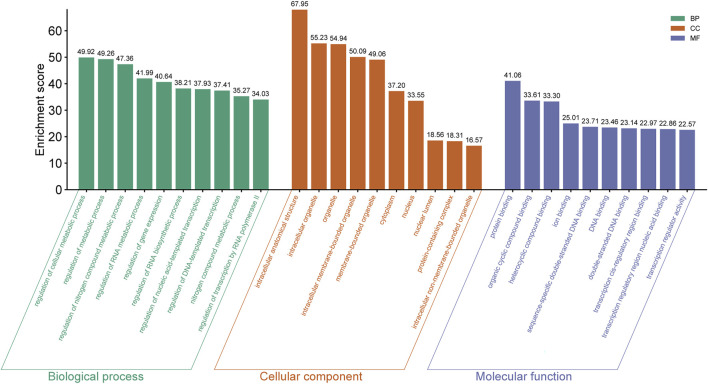
The top ten GO terms enriched with host genes in the BP, CC and MF categories (ranking by P Value) are listed.

In mouse pancreatic exosomes, the TGs of DE miRNAs were found to be substantially correlated with 1937 biological process outcomes. The top ten results, ranked by p-value, are as follows: regulation of cellular metabolic process, regulation of metabolic process, regulation of nitrogen compound metabolic processes, regulation of RNA metabolic process, regulation of gene expression, regulation of RNA biosynthetic process, nitrogen compound metabolic process, regulation of nucleic acid-templated transcription, regulation of DNA-templated transcription, and regulation of transcription by RNA polymerase II (Figure 4).

TGs of DE miRNAs were discovered to be substantially correlated with 240 outcomes of cellular components in mouse pancreatic exosomes. Ranked by p-value, the cellular components that were found to be affected by the predicted TGs included intracellular anatomical structure, intracellular organelle, organelle, intracellular membrane-bounded organelle, membrane-bounded organelle, cytoplasm, nucleus, nuclear lumen, protein-containing complex and intracellular non-membrane-bounded organelle (Figure 4).

It was discovered that there was a substantial correlation between the molecular functions of 258 terms and the TGs of DE miRNAs in mouse pancreatic exosomes. The top ten results by leveling p-valve were protein binding, heterocyclic compound binding, ion binding, sequence-specific double-stranded DNA binding, DNA binding, transcription regulatory region nucleic acid binding, double-stranded DNA binding, transcription cis-regulatory region binding, and transcription regulator activity (Figure 4).

### Enrichment of meaningful pathways of TGs of DE miRNAs

**FIGURE 5:**
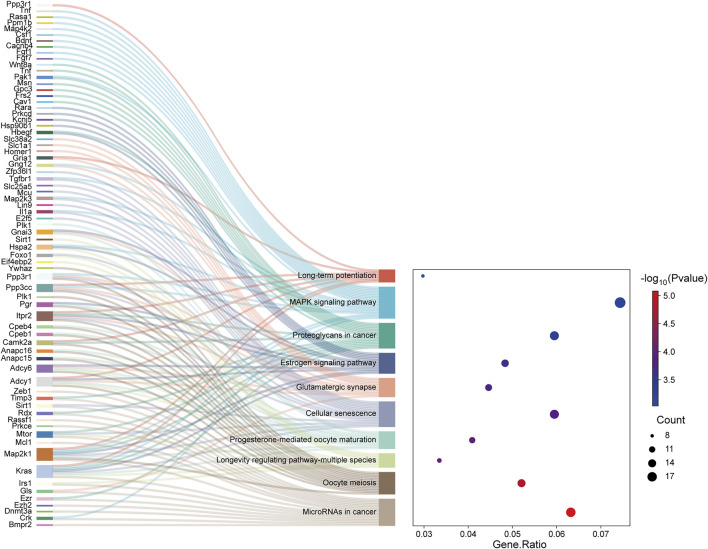
The Sankey map showed that DE genes among the transcripts are involved in the enrichment pathway obtained by KEGG pathway enrichment analysis. The dot plot showed the ratio of the number of DE gene entities to the total number of DE gene entities in each enriched pathway (FDR-adjusted P-value <0.05).

The TGs of the most important exosome-derived miRNAs were found to be abundant in the following pathways, according to KEGG pathway analysis: MicroRNAs in cancer, Oocyte meiosis, Longevity regulating pathway-multiple species, Cellular senescence, Progesterone-mediated oocyte maturation, Glutamatergic synapse, Estrogen signaling pathway, Proteoglycans in cancer, MAPK signaling pathway and Long-term potentiation (Figure 5).

### Validation of exosome-derived DE miRNAs

**FIGURE 6:**
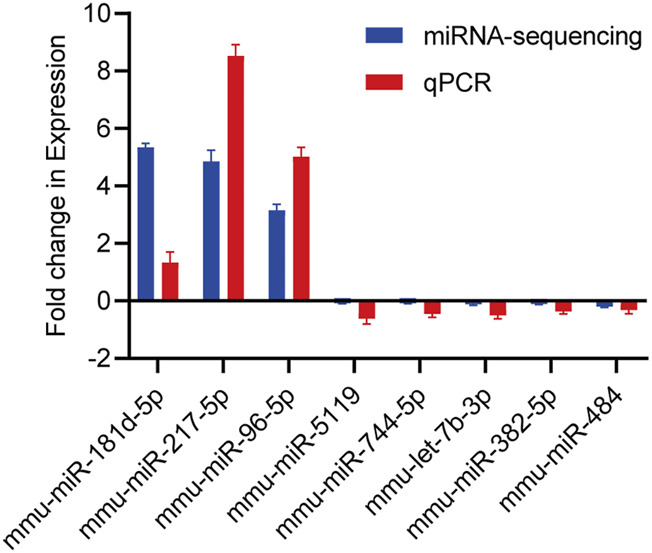
Validation of candidate miRNAs by miRNA-sequencing and qPCR. The upwards represents upregulation, while the downwards represents downregulation. Data are presented as mean ± SD from technical triplicates. Bar plots represent the normalized expression levels of these miRNAs, showing trends consistent with the miRNA-sequencing data. Statistical significance was determined using Student’s t-test (p < 0.05). Quantitative PCR was performed for each cDNA sample in technical triplicate.

For 8 exosome-derived miRNAs with notably varied expressions, we employed qPCR to demonstrate the expression levels. The results showed that the expression trends of 8 miRNAs identified by qPCR (mmu-miR-181d-5p, mmu-miR-217-5p, mmu-miR-96-5p, mmu-miR-5119, mmu-miR-744-5p, mmu-let-7b-3p, mmu-miR-382-5p and mmu-miR-484) was similar to that of sequencing data, as visually summarized in Figure 6. The reliability of our miRNA-seq data was shown by this outcome.

## Discussion

To the best of our knowledge, this work reported the expression profile of exosomal miRNAs in a mouse model of pancreatic injury caused by OSA for the first time. In addition, our study showed some potential targets and pathways for exosomal miRNA involvement in pancreatic injury. More importantly, this study tried to characterize the consequences of exosome-derived miRNAs in OSA-related diabetes mellitus and widened our understanding of their roles.

Recurrent apnea or hypopnea during sleep, which lowers blood oxygen saturation, is the hallmark of OSA (Yeghiazarians et al., 2021). The risk of several comorbidities, including diabetes mellitus, depression, dementia, cardiovascular and cerebrovascular disease, and even some types of cancer, may rise with severe OSA (Hunyor and Cook, 2018; Vanek et al., 2020). Notably, a number of studies are discovering that OSA is thought to contribute to pancreatic damage (Wang et al., 2019). Nonetheless, there are still certain challenges in researching the connection between pancreatic damage and OSA. First, it is difficult to pinpoint a single mechanism of OSA induction since, individuals with pancreatic damage caused by OSA often have many risk factors, such as obesity, diabetes mellitus, and smoking. Moreover, because pancreatic damage, such as chronic pancreatitis, often takes years to become clinically visible, it is difficult to determine changes in pancreatic damage status in OSA patients. Due to the limitation of research on the macroscopic causal relationship between OSA and pancreatic injury, scientists are now looking at novel targets in the fields of molecular biology and epigenetics.

Exosomes are small molecular cellular vesicles, varying in size from 30 nm to 150 nm, and are produced by most body cells (Yang et al., 2020; Hu et al., 2020; Whitford and Guterstam, 2019). Exosomes can transport mRNAs, miRNAs, ncRNAs, proteins, lipids, and metabolites (Mori et al., 2019; Hu et al., 2020; Isaac et al., 2021). According to research by Tan, S., et al., tumor-derived exosomal miRNAs cause matrix reprogramming in the tumor microenvironment, which creates a microenvironment conducive to tumor development, metastasis, immunological evasion, and treatment resistance (Tan et al., 2020). In addition, Nahand et al. have shown that exosomal miRNAs are linked to the viral infection process (Nahand et al., 2020). But it's still unknown how exosome-derived miRNAs affect the way OSA causes pancreatic damage in mice.

In the current work, we sequenced the samples to better understand the expression profile of exosomal miRNAs in mice with pancreatic damage produced by CIH. We discovered 33 downregulated miRNAs, 55 newly predicted miRNAs, and 3 upregulated miRNAs. After that, we used qPCR to verify the sequencing data, and concluded that the change trend in the sequencing data was in line with the qPCR data. It is noteworthy that the specific biological roles of exosomal miRNAs in mouse model of CIH-induced pancreatic damage tissue and associated target genes remain elusive.

Beyond their potential regulatory roles, the newly predicted miRNAs may hold diagnostic value and play roles in endocrine regulation (Singh et al., 2024). Their specific presence in exosomes derived from injured pancreatic tissue positions them as potential non-invasive biomarkers. Supported by high miRDeep2 scores, these miRNAs are high-priority candidates for future studies aimed at verifying their diagnostic utility and endocrine regulatory functions in OSA-related pancreatic pathophysiology. To investigate the biological activities of certain targets and pathways, we performed KEGG and GO analyses for the target genes. According to BP results, regulation of cellular metabolic process is the most enriched term. Le’s research has demonstrated that colonization of the intestinal microbiota in patients with hypertriglyceridemia pancreatitis (HTGP) can recruit neutrophils and increase the formation of neutrophilic extracellular traps (NETs), thus exacerbating pancreatic injury and systemic inflammation (L et al., 2023). According to Wang, Y., et al., strong antioxidant tempol was found to inhibit IH-induced pancreatic apoptosis by upregulating Bcl-2 levels and downregulating Bax and cleaved caspase-3 levels (Wang et al., 2019). However, Studies on the other potential targets and molecular markers linked to pancreatic injury caused by OSA are not entirely clear.

KEGG pathway analysis identified several enriched signaling pathways, with the top five being microRNAs in cancer, progesterone-mediated oocyte maturation, oocyte meiosis, longevity regulating pathway-multiple species, and cellular senescence. While pathways such as 'MicroRNAs in cancer,’ 'Cellular senescence,’ and the 'MAPK signaling pathway’ have well-documented roles in apoptosis, inflammation, and insulin resistance, the enrichment of others including 'Oocyte meiosis’ and 'Progesterone-mediated oocyte maturation’ requires careful interpretation. This likely does not reflect a direct physiological role in oocyte function but rather implicates shared core cell cycle regulators (e.g., CDK1, PLK1) and conserved signaling molecules across biological processes (Das and Arur, 2022). *Kras*, *Gls*, *Bmpr2*, *Irs1*, *Foxo1*, and *Map2k1* were related to the pathways mentioned above. Dai’s study showed that extracellular KRAS^G12D^ was packaged as exosomes and then was taken up by macrophages via an AGER-dependent mechanism, thus promoting the polarization of pancreatic tumor-associated macrophages (Dai et al., 2020). Li’s work revealed that the decreased expression of LncRNA *MEG3* (*MEG3*) and *FOXO1* can lead to autophagy dysfunction and pyroptosis of pancreatic β-cells (Li et al., 2022). In addition, the decreased expression of the Irs1 gene has an anti-apoptotic effect on the pancreas and INS-1 cells of diabetic rats, and helps to protect the pancreatic tissue of rats (Li et al., 2017). Notably, it is unknown how these genes function in pancreatic injury caused by OSA. The significant enrichment of these pathways in our CIH pancreatic injury model suggests a profound dysregulation of fundamental cellular processes, such as cell cycle control and survival signaling. Therefore, the subsequent discussion will focus on pathways with more established relevance to pancreatic pathophysiology. The MAPK pathway was of particular interest due to its established crucial role in the pathophysiology of pancreatic injury. In acute pancreatitis (AP), p38 MAPK is activated early and promotes inflammation, apoptosis, and tissue damage. Inhibition of p38 MAPK (e.g., by SB203580) has been shown to alleviate pancreatic inflammation and associated acute renal injury in AP models (Bläuer et al., 2021; Zhang et al., 2021). In the context of chronic pancreatitis (CP), MAPK signaling drives inflammatory cell infiltration and fibrosis, a phenomenon underscored by the therapeutic effects of agents like Dachaihu Decoction (DCHD), which act through modulation of the MAPK pathway (Li et al., 2025).

To investigate potential meaningful targets for exosomal miRNAs in mouse pancreatic tissue induced by OSA, we performed a ceRNA analysis and mapped a CNC network. In the upregulated miRNAs, such as mmu-miR-181d-5p, Li’s study indicated that inhibiting the expression of the *YTHDC2* gene could effectively prevent ferroptosis (Li et al., 2024). *YTHDC2* is also represented in our CNC network map, and the relationship between this gene and pancreatic injury in mice is unclear. In the downregulated miRNAs, such as mmu-let-7b-3p, *FOXD3* is seen to be associated with this miRNA. According to research by Facuz et al., a deficiency in Foxd3 results in an increase in the proliferation of insulin-expressing cells, an absence of neural crest cells, and insulin-positive areas (Faucz et al., 2023). They discovered that while the quantity of cells expressing insulin rose, there was a notable hindering of β-cell maturation. In our study, the relationship between abnormal *FOXD3* expression and the downregulation of this miRNA in mouse pancreatic injury tissue induced by OSA needs to be further investigated.

However, there are some limitations in our study. First, the small sample size (n = 3 per group) in this exploratory study limits the statistical power and generalizability of our findings. Future studies with larger cohorts and *a priori* power calculations are warranted to validate these results. Second, the current study lacks functional validation of the identified DE miRNAs. While our sequencing and bioinformatics analyses provide a crucial first map of the altered miRNA landscape, the proposed roles of miRNAs such as mmu-miR-96-5p and mmu-let-7b-3p remain speculative. Future studies employing *in vitro* models (e.g., miRNA mimics/inhibitors in pancreatic β-cell lines like MIN6 or INS-1 exposed to intermittent hypoxia) or *in vivo* approaches in suitable models are essential to definitively establish the causal roles of these miRNAs in CIH-induced pancreatic injury. Third, our understanding of the expression profile of exosome-derived miRNAs in OSA-induced mice pancreatic damage tissue is limited, and more epigenetic research is required to fully understand the function of individual miRNAs. Fourth, HE pathological scores should be made for pancreatic tissues in each group, which will make the results more convincing. Lastly, it should be noted that while our study utilizes a well-established CIH model to simulate the hypoxic stress of obstructive sleep apnea, clinical OSA is a multifactorial condition that also involves sleep fragmentation, physical airway obstruction, and intrathoracic pressure changes. The CIH model primarily reproduces the intermittent hypoxia component, which is a key driver of metabolic and pancreatic dysfunction in OSA. Therefore, future studies incorporating other OSA features may provide a more comprehensive understanding of the disease pathophysiology.

In addition, a key limitation of this study is the lack of direct correlation between the identified exosomal miRNA signatures and functional metabolic parameters (e.g., insulin levels, glucose tolerance) or quantitative histology data (e.g., β-cell mass, apoptosis) obtained from the same cohorts. Although our prior work confirms that this chronic intermittent hypoxia (CIH) model induces pancreatic injury and metabolic dysfunction (Chen et al., 2023), future studies must directly integrate these molecular profiles with phenotypic outcomes. Such integration is essential to establish causal links between specific miRNAs and the manifestations of pancreatic damage, ultimately validating their roles as key mediators or biomarkers.

In conclusion, our research revealed the miRNA expression profile of exosomes in mouse pancreatic damage tissue caused by OSA for the first time, offering fresh perspectives for future research on related targets.

## Data Availability

The datasets generated and analysed during the current study are available in the GEO repository, https://www.ncbi.nlm.nih.gov/geo/query/acc.cgi?acc=GSE279909, enter token into the box is efehusmcblyprof.
